# Clinical and patient‐centered outcomes post non‐surgical periodontal therapy with the use of a non‐injectable anesthetic product: A randomized clinical study

**DOI:** 10.1111/jicd.12446

**Published:** 2019-07-28

**Authors:** Simone Marconcini, Marilyn Goulding, Giacomo Oldoini, Chiara Attanasio, Enrica Giammarinaro, Annamaria Genovesi

**Affiliations:** ^1^ Tuscan Stomatologic Institute Camaiore Italy; ^2^ Dentsply Vaughan Canada

**Keywords:** anesthetic, clinical, decontamination, gingival, visual analog scale

## Abstract

**Aim:**

The aim of this study was to determine the impact of different full‐mouth decontamination (FMD) protocols on the effectiveness of an intrapocket anesthetic gel in periodontal maintenance patients.

**Methods:**

Patients undergoing the periodontal maintenance program and with the need for FMD participated in this study. Patients were randomly allocated to non‐surgical periodontal therapy (NSPT) with either a preparatory 15‐day decontamination phase, including chlorhexidine mouth rinse and domiciliary hygiene instructions (modified FMD: test group), or without it (FMD: control group). In both groups, NSPT was performed with the aid of a non‐injectable anesthetic gel. Clinical and patient‐related outcomes were recorded during a 6‐month follow‐up period.

**Results:**

Sixty patients completed the 6‐month study. Both groups experienced relevant clinical improvements after NSPT, but the test group showed a significant change in periodontal parameters already after the initial 15‐day preparatory period, and overall significantly better results in periodontal outcomes when compared with the control group at the last 6‐month follow up: the gingival index was 2.07 ± 1.25 in the control group and 1.13 ± 0.51 in the test group. Less pain and dental‐related anxiety were perceived by patients in the test group showing a 6‐month mean visual analog scale of 2.13 ± 1.25 in the control group and 1.13 ± 0.83 in the test group.

**Conclusion:**

The present study suggested that the modification of the standard FMD could improve the clinical efficacy of non‐injectable anesthetic, along with patients’ short‐ and mid‐term appreciation and compliance.

## 1 INTRODUCTION

It has been demonstrated that the core treatment of periodontal disease remains scaling and root planing (SRP), namely the non‐surgical removal of subgingival bacterial deposits.^1^, ^2^ Numerous influential systematic reviews have reported consistent clinical improvement in patients with periodontitis after complete subgingival debridement, which resulted in effectively reducing probing pocket depth (PPD) and in improving the clinical attachment level (CAL).^3^, ^4^, ^5^ Non‐surgical periodontal therapy (NSPT) includes manual and ultrasonic instrumentation in conjunction with supragingival plaque control.^6^ It must be said that the management of some severe clinical scenarios may still require surgical intervention too.

There are 2 main ways to deliver NSPT: quadrant and full‐mouth SRP. Quadrant scaling consists of several sessions of SRP alone or in combination with adjunctive antimicrobial therapy at 1‐3‐week intervals.^7^ Full‐mouth scaling consists of 1‐stage (within 24 hours) SRP. The approach was first introduced by Quirynen et al and known as full‐mouth decontamination (FMD)^8^; the stated objective was to provide patients with accelerated periodontal therapy, thereby avoiding the potential interim translocation of pathogens, and preventing the reinfection of previously treated sites by microorganisms from untreated pockets or within other intraoral niches.

Recent data suggested that NSPT performed fully within 24 hours may induce greater disturbance of systemic inflammation when compared with quadrant scaling.^9^ To overcome this issue, different modifications to the original FMD protocol have been proposed. The modified FMD (MFMD) introduced by Genovesi et al begins with a purely instructional/motivational session and continues with a 2‐week at‐home chlorhexidine (CHX) regimen coupled with coached oral‐hygiene measures in preparation for SRP, purportedly reducing the risk of patients’ discomfort and systemic inflammation.^10^ Different studies have assessed the benefits of combining full‐mouth debridement with antimicrobial agents and antiseptic rinses.^11^ The efficacy of CHX rinsing paired with SRP has been questioned, as some studies have failed to show additional benefits even after extended use. As a matter of fact, after SRP, CHX should be time‐limited and soon replaced by proactive therapy. The use of CHX for longer than 15 days is unnecessary because of undesirable side‐effects such as staining, taste disruption and/or local microbiome derangement.^12^

Regardless of the protocol, SPT has often been associated with pain and discomfort of the patient.^13^ Notably, the response to pain varies greatly from person to person and it is often modulated by the level of local inflammation and by individual patient arousal (level of anxiety and apprehension).^14^ Nevertheless, local injection anesthetic for pain control before NSPT is not often welcomed by all patients, preventing actual delivery of FMD in some cases.

A large study based on an international telephone survey found that participants considered local anesthetic injection as painful, and 1/3rd would rather accept some discomfort during the periodontal procedure vs being subjected to an injection.^15^ Clinical efficacy and patients’ acceptance of anesthetic gels prior to SRP is described in the literature.^16^ Patients claimed to be willing to pay an additional amount of money to cover the expense of the anesthetic gel. The greatest advantage of non‐injectable gels is reduced postoperative numbness and discomfort when compared with injectable anesthetic. Furthermore, it is reported that patients are more willing to return for recall visits if the gel is available at the office.^17^ Finally, non‐injectable anesthetic is time‐efficient, thus being in both the patient's and clinician's interest. However, little is known about the ideal working conditions of this medicament.

The null hypothesis of the present study would have been that there were no differences in terms of gel anesthetic effectiveness depending on the type of FMD protocol used. The aim was to investigate whether a preparatory period aiming at reducing the initial local inflammation could have influenced the perceived performance of a non‐injectable anesthetic (Oraqix^®^; Dentsply, Konstanz, Germany).

## 2 MATERIALS AND METHODS

### 2.1 Study design

A randomized, prospective, parallel study design was adopted. Eligible patients were recruited from those attending the Tuscan Stomatologic Institute for standard non‐surgical periodontal treatment (NSPT). The local ethics committee gave the approval for this study.

### 2.2 Patient selection criteria

The inclusion criteria were as follows: age of 18 years or older; patients scheduled for NSPT with injection anesthetic; and patients of good general health. Subjects exhibiting 1 of the following criteria were excluded from the study: history of hypersensitivity to lidocaine, prilocaine or local anesthetic of the amide type, or to any excipients in the preparation; severe hepatic disease; diabetes; congenital or idiopathic methemoglobinemia; pregnancy or lactation; and having received periodontitis treatment within the 6 months prior to start of the study.

Those satisfying the inclusion criteria were asked to fill out an anamnestic questionnaire, covering participants’ age, gender, tobacco consumption and continuative drug intake. All eligible participants were assigned a consecutive study number. Patients were randomly allocated to 1 of 2 possible groups of treatment through a computer‐generated list (see below). Sample size estimation was calculated to achieve a significant difference in the intra‐ and intergroup analysis in periodontal parameters and patient‐related outcomes.^20^

### 2.3 Treatment groups

#### 2.3.1 Group 1 or test group

At baseline, patients allocated to the test group received standardized instructions regarding the home‐based disinfection protocol. The 15‐day home pretreatment program (preparatory period) included mechanical plaque control consisting of tooth‐brushing with toothpaste and interdental cleaning devices, tongue brushing with a 1% CHX gel, followed by 0.2% CHX solution mouth rinse, twice a day for 2 weeks (Plakout Active 0.20%; Polifarma Benessere, Rome, Italy). After 2 weeks, full‐mouth non‐surgical periodontal treatment (FMSRP) was performed with the use of Oraqix on each quadrant in 1 sitting.

#### 2.3.2 Group 2 or control group

At baseline, periodontal non‐surgical treatment was performed immediately, with no attempts to reduce existing conditions prior to therapy. After SRP, patients were instructed exactly as per the test group. The standard non‐surgical FMSRP was delivered following the application of Oraqix on each quadrant, as per the manufacturer's instructions, in 1 sitting,

### 2.4 Follow up

Patients were evaluated at baseline, and at 15 days, 30 days, 90 days and 6 months after NSPT.

### 2.5 Periodontal treatment

In this study, NSPT consisted of removal of plaque and calculus utilizing sharp mini‐curettes and ultrasonic inserts until the surfaces were hard and smooth. Before SRP, patients in both groups received Oraqix, a topical anesthetic agent used in periodontal treatments for pain control. It consists of a eutectic mixture of 5% lidocaine and prilocaine (each gram contains 25 mg lidocaine and 25 mg prilocaine). The teeth were isolated with cotton rolls or lip separators and the pockets were dried with paper points in order to soak up the crevicular fluid, maximizing the substantivity of the gel applied. Oraqix was placed inside the periodontal pockets from distal to mesial using a plunger/applicator designed for such purpose. When re‐anesthetic was needed, the clinician placed more Oraqix on the sensitive area (not to exceed 5 vials in total) and recorded the quantity and number of vials used.

No analgesic or anti‐inflammatory drugs were prescribed in this study in order to evaluate the effects of treatments exclusively.

### 2.6 Clinical assessment

The full‐mouth plaque index (FMPI), full‐mouth bleeding on probing (FMBS), gingival index (GI) and probing depth to the nearest millimeter were recorded using a PCP‐UNC 15 probe (Hu‐Friedy). 3rd molars were excluded.

### 2.7 Psychological assessment

Before clinical examination, 2 psychological instruments were administered to the participants. The dental anxiety score (proposed by Corah in 1969)^18^ is a survey of 4 questions directly related to dental anxiety: (a) “If you were to go to the dentist tomorrow, how would you feel?”; (b) “While you wait in the office, how do you feel?”; (c) “While you are in the dentist's chair waiting for him/her to take the drill to start work on your teeth, how do you feel?”; and (d) “You are in the dentist's chair to clean your teeth. How do you feel while the dentist takes the instruments to clean your teeth?”. Each question has 5 choices of answer, and the final score can range 4‐20. The dental fear score (proposed by Kleinknecht and Klepac in 1973)^19^ is an instrument to assess dental fear and attempts to avoid treatment. It is a Likert‐like questionnaire consisting of 20 items that assess issues related to the avoidance of treatment, somatic visceral excitement and how much fear is caused by the stimuli associated with dental treatment. The score for each question ranges from 1 (little fear) to 5 (very afraid), the total range being 20‐100, with positive correlation to increasing dental fear.

Within 5 minutes after treatment, patients were presented the following question: “How much pain did you feel during the SRP procedure?”. Each patient received a postoperative pain sheet with a visual analog scale (VAS).^21^ The VAS used was a continuous scale comprised of a horizontal line, measuring 100 mm in length, anchored by 2 verbal descriptors, 1 for each symptom extreme: “no pain” at the far left (score of 0) and “pain as bad as it could be” or “worst imaginable pain” at the far right (score of 100). The respondent was instructed to mark the point that represented the pain intensity.

### 2.8 Statistical analysis

Descriptive, difference and correlation analysis was performed (R version 3.5.1 [2018‐07‐02] ‐ "Feather Spray”). Each variable of interest was assigned to the appropriate statistical test according to its nature: independent/dependent, continuous/nominal/time‐to‐event and normal/non‐normal. A repeated‐measures ANOVA design was chosen for the factorial multivariate analysis. The association of continuous non‐parametric variables was checked with kernel regression. Kernel regression was used to estimate the association of several variables for postoperative pain (VAS). Inferential statistics was performed using the method of Noguchi et al; several tests for the relative treatment effects with global or patterned alternatives for the F1‐LD‐F1 design were applied for testing group (treatment) and time effects, and their interactions.^22^ Moreover, pairwise comparisons of the groups, patterned interactions and patterned group effects were tested using this function.

## 3 RESULTS

### 3.1 Demographic details

Table 1 presents the demographic characteristics of the cohort. A total of 60 patients with a mean age of 47 years completed the follow up. There were no significant differences between groups with respect to gender, age and smoking (*P *> .05).

**Table 1:** Demographic data

	FMD	MFMD
Sample size	30	30
Age (years)	47.5 ± 17.9	47.6 ± 12.3
Age range (years)	31.0‐68.4	38.8‐65.8
Gender ratio, M/F	12/18	14/16
Smoking habit, Y/N	20/10	21/9

Abbreviations: FMD, full‐mouth decontamination; MFMD, modified full‐mouth decontamination.

### 3.2 Anesthetic vial proportion

**Figure 1:**
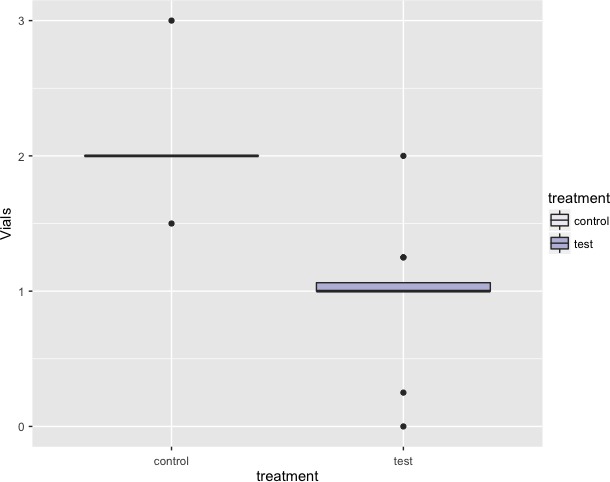
Median quantity of anesthetic vials needed at second recall

The non‐injectable anesthetic product quantity used was expressed in vial quarters. A mean of 2 vials were necessary for patients in the control group whereas 1 vial was sufficient for patients in the test group (Figure 1)*.*

### 3.3 Clinical assessment

**Figure 2:**
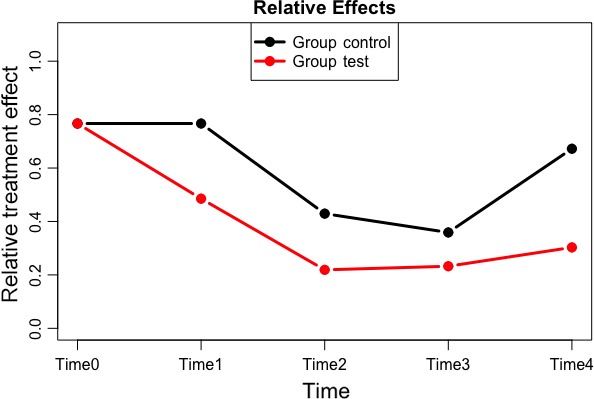
Plot of the relative treatment effect for gingival index

**Figure 3:**
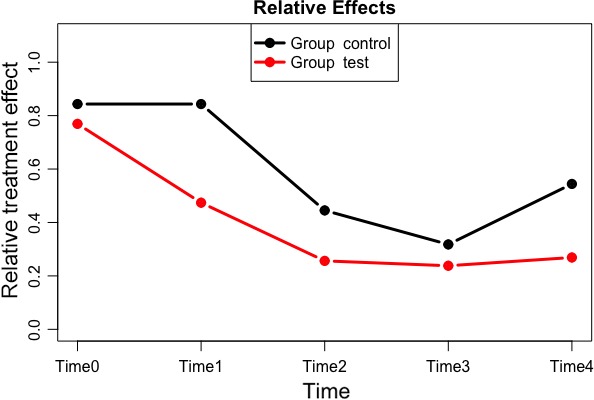
Plot of the relative treatment effect for full‐mouth plaque index

**Figure 4:**
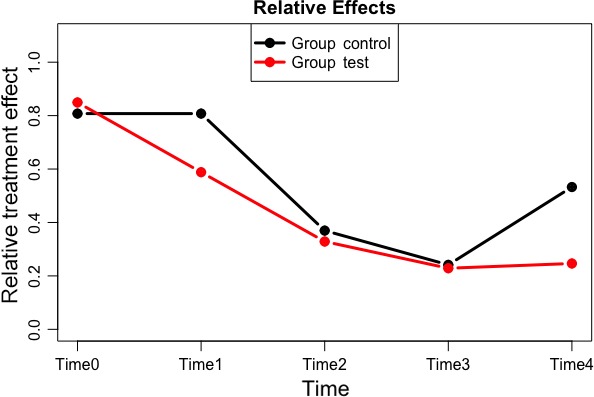
Plot of the relative treatment effect for full‐mouth bleeding score

Mean and standard deviations for each parameter explored are presented in Tables 2 and 3. All clinical parameters significantly improved (*P* < .001) over the 6‐month period of the study. There was a significant interaction between treatment groups and time on the indices of periodontal inflammation demonstrated with each test within the f1.ld.f1 function. Figures 2, 3 and 4 report the plots of the relative treatment effect for GI, FMPI and FMBS, respectively. Patients in the test group showed the most benefit from full‐mouth NSPT when compared with patients assigned to the control group (*P* < .0001). For the sake of simplicity, we are presenting raw numbers only for the ANOVA test (Table 4).

**Table 2:** Intergroup comparison of mean, standard deviation and median of main periodontal parameters explored

	Baseline	3 months	6 months
Gingival index			
FMD	2.40 ± 0.50 2	1.27 ± 0.59 1	2.07 ± 0.25 2
MFMD	2.40 ± 0.50 2	0.93 ± 0.45 1	1.13 ± 0.51 1
Full‐mouth plaque index (%)			
FMD	77.1 ± 18.0 84	22.1 ± 4.59 23	35.1 ± 7.23 35
MFMD	69.7 ± 24.5 74	19.0 ± 9.45 16	19.3 ± 11.0 20
Full‐mouth bleeding score (%)			
FMD	76.6 ± 11.7 80	21.1 ± 5.93 22	39.0 ± 9.62 40
MFMD	81.9 ± 20.5 90	20.3 ± 9.22 20	21.3 ± 9.68 20

Abbreviations: FMD, full‐mouth decontamination; MFMD, modified full‐mouth decontamination.

**Table 3:** Intergroup comparison of mean and standard deviation of patient‐related outcomes

	Baseline	6 months
VAS		
FMD	3.80 ± 1.32	2.13 ± 1.25
MFMD	3.60 ± 1.88	1.13 ± 0.83
DAS		
FMD	14.2 ± 3.66	10.2 ± 4.91
MFMD	16.8 ± 4.98	10.0 ± 2.67
DFS		
FMD	38.3 ± 10.5	35.3 ± 8.56
MFMD	41 ± 7.75	24.2 ± 8.59

Abbreviations: DAS, dental anxiety score; DFS, dental fear score; FMD, full‐mouth decontamination; MFMD, modified full‐mouth decontamination; VAS, visual analog scale.

**Table 4:** ANOVA relative to group, time and their interaction on the clinical parameters explored.

ANOVA			
	Statistic	*df*	*P*
Gingival index			
Group	31.551662	1.00000	1.942041e‐08
Time	54.856323	3.27906	1.734952e‐38
Group:time	6.930997	3.27906	6.448493e‐05
Full‐mouth plaque index			
Group	18.554514	1.000000	1.651137e‐05
Time	71.194278	2.922897	6.288929e‐45
Group:time	5.779011	2.922897	6.828015e‐04
Full‐mouth bleeding score			
Group	6.569763	1.00000	1.037258e‐02
Time	95.902905	2.74252	5.318402e‐57
Group:time	7.612239	2.74252	8.132877e‐05

### 3.4 Patient‐related outcomes

There was a significant interaction between treatment groups and time on patient‐related outcomes (VAS, DAS, DFS) demonstrated with each test within the f1.ld.f1 function. Patients in the test group showed the lowest scores for pain, dental anxiety and treatment avoidance over time when compared with the control group (*P* < .0001) (Table 5).

**Table 5:** ANOVA relative to group, time and their interaction on patient‐related parameters

ANOVA			
	Statistic	*df*	*P*
VAS			
Group	3.351446	1	6.714599e‐02
Time	68.298216	1	1.405481e‐16
Group:time	2.065731	1	1.506427e‐01
DAS			
Group	1.010713	1	3.147322e‐01
Time	35.148679	1	3.054686e‐09
Group:time	6.966746	1	8.303834e‐03
DFS			
Group	0.3391223	1	5.603363e‐01
Time	18.4294241	1	1.763143e‐05
Group:time	6.5823520	1	1.029947e‐02

Abbreviations: DAS, dental anxiety score; DFS, dental fear score; VAS, visual analog scale.

In the kernel regression univariate analysis, postoperative pain was significantly associated with severe periodontal inflammation (*r* ^2^ = .3492695) and dental anxiety (*r* ^2^ = .6482695).

## 4 DISCUSSION

Non‐surgical periodontal treatment by means of SRP is considered to be the basis of periodontal maintenance and therapy.^23^ The results of the present study suggested that the MFMD is a valid way to approach SRP and that it is associated with great patient acceptance. Patient‐related outcomes turned out to be better for the test group (MFMD) than the control group (FMD), regardless of the amount of anesthetic gel used. Patients in the test group, those who accomplished the 15‐day preparatory period before SRP, required significantly less anesthetic gel than patients in the standard control group. This finding may be due to the preliminary reduction in local inflammation achieved with the MFMD, possibly unlocking the full potential of the topical anesthetic. Additionally, MFMD encouraged the patients’ active participation in therapeutic goal accomplishment.

The main findings of our study are in line with those of Derman et al who showed that the effectiveness of local anesthetic in gel was related to pocket probing depth.^24^

It has been suggested by Schirmer et al that post‐NSPT pain is associated with dental anxiety and baseline inflammation.^14^ In their study, the prevalence of pain after NSPT with local anesthetic was higher for patients with severe periodontal inflammation, defined as the presence of at least 4 sites with a probing depth of 6 mm or more.

The classic 2004 cross‐over study by Sekino et al demonstrated that CHX used as a mouth rinse during the preparatory period significantly delayed the plaque formation, as well as decreasing the counts of salivary and tissue bacteria.^25^ It could be advocated that the benefits of the patients’ preparation by means of motivation/instruction and chemical detoxification go beyond the mere antimicrobial effect. In fact, data suggest that even though the microbiota is almost suppressed by CHX, the microbiota spontaneously return to that observed after mechanical means alone.^26^ This reinforces the notion that the microenvironment is critical in controlling the actual bacterial composition of the local microbiome, and that the microbiota will tend to return to that characteristic of the specific individual, once antimicrobial means are withdrawn.^27^

The patients’ perceived experience is a fundamental component of the global effect of full‐mouth NSPT. In fact, the negative experience of dental anxiety results in greater avoidance of, and delay in, dental hygiene appointments, resulting in deteriorated oral health with higher treatment needs and costs, with the potential loss of further patient attendance at the dental office.^28^ The modified FMD supports the modern tendency towards patient‐centered approaches with the patient becoming a proactive part of a long‐term preventive therapy. Therefore, the true benefit of the 15‐day preparatory period is likely the impetus of the patients’ understanding of, and compliance with, therapy.

Sekino et al rejected the hypothesis that the FMD protocol could generate greater anxiety and pain, and that the severity of periodontitis increases pain, fear and anxiety scores.^25^ Furthermore, the severity of periodontal clinical parameters did not influence the DAS and DFS scores. The findings of the present study are similar, suggesting that the full‐mouth approach does not add any significant change to the patients’ anxiety level if compared with the per‐quadrant procedure.

The use of a local anesthetic agent by the dental hygienist is a cost‐ and time‐saving procedure: the MFMD approach combined with the use of Oraqix gel allowed savings of approximately 20 minutes per session when compared with the average time needed by MFMD combined with conventional anesthetic injection, which requires greater onset time and logistical effort to arrange with the dentist (data drawn from previous clinical records).

A limitation of the present study may relate to the psychological factors being provided from a self‐reported questionnaire, as patients may be inconsistent in expressing their personal views about personal health.^29^ Furthermore, local inflammation was assessed with indirect measures such as clinical and patient‐reported parameters; it would be interesting to test the present protocol including local and systemic oxidative stress and pro‐inflammatory marker expression immediately after SRP. Larger studies with stratification of patients according to periodontal disease severity would be useful to back up the present findings.

In conclusion, this study suggested that the MFMD protocol combined with the application of an anesthetic gel immediately before SRP allows for time‐efficient non‐surgical therapy with overall improved clinical parameters, less use of medication, reduced dental anxiety and greater patient acceptance.

## CONFLICT OF INTEREST

None declared.
